# Dissociation of Pupillary Post-Illumination Responses from Visual Function in Confirmed *OPA1* c.983A > G and c.2708_2711delTTAG Autosomal Dominant Optic Atrophy

**DOI:** 10.3389/fneur.2015.00005

**Published:** 2015-02-04

**Authors:** Claus Nissen, Cecilia Rönnbäck, Birgit Sander, Kristina Herbst, Dan Milea, Michael Larsen, Henrik Lund-Andersen

**Affiliations:** ^1^Department of Ophthalmology, Glostrup Hospital, University of Copenhagen, Copenhagen, Denmark; ^2^Singapore National Eye Centre, Singapore Eye Research Institute, Duke-NUS Graduate Medical School Singapore, Singapore, Singapore; ^3^Angers University Hospital, Angers, France

**Keywords:** autosomal dominant optic atrophy, pupillary light reflex, melanopsin, intrinsically photosensitive retinal ganglion cells, ipRGC

## Abstract

**Purpose:** To test whether the melanopsin-containing, intrinsically photosensitive retinal ganglion cells (ipRGCs), as evaluated by examination of the pupillary light reflex (PLR), are preserved in genetically confirmed autosomal dominant optic atrophy (ADOA).

**Method:** Twenty-nine patients with either the c.983A > G (*n* = 14) or the c.2708_ 2711delTTAG mutation (*n* = 15) were examined with monochromatic pupillometry, using isoluminant (300 cd/m^2^), red (660 nm) or blue (470 nm) light, optical coherence tomography, automated visual field analysis, and with determination of best corrected visual acuity (BCVA). Since we examined two different mutations, initially we compared all outcome variables between the two, and finding no statistically significant difference, pooled them.

**Results:** Despite a poor BCVA (56 letters, ETDRS) in the ADOA patients, their post-illuminatory pupil responses did not differ significantly from those of healthy controls (blue, *p* = 0.45, red, *p* = 0.49, *t*-test), and no statistically significant effect was noted of peripapillary retinal nerve fiber layer thickness, ganglion cell-inner plexiform layer thickness, or age.

**Conclusion:** The PLR to blue light of high luminance (300 cd/m^2^) was preserved in both c.983A > G and c.2708_2711delTTAG ADOA despite severe visual loss and optic nerve atrophy. The study confirms, in a large sample of two genetically homogenous groups, that the ipRGCs are spared in ADOA.

## Introduction

The intrinsically photosensitive retinal ganglion cells (ipRGC) play a key role in the physiology of the pupillary light reflex (PLR), as well as in other non-image-forming (NIF) light responses, including entrainment of circadian rhythms and regulation of secretion of the hormone melatonin (1–4). The intrinsic photosensitivity of the ipRGCs is due to melanopsin, an opsin, which exhibits maximal absorption to blue (480 nm) light (5–7). IpRGCs respond directly to light and in addition receive synaptic input from rods and cones (1). The contribution of melanopsin (the intrinsic response) to the PLR is best evaluated by monochromatic pupillometry, measuring the sustained post-illumination pupillary response, after blue light stimulation (8), while the synaptic contribution from cones is measured during stimulation with red light (660 nm) of high luminance (300 cd/m^2^).

Previous pupillometry studies have shown abnormal pupillary responses in anterior ischemic optic neuropathy and glaucoma (9–11), whereas responses were normal in Leber hereditary optic neuropathy (LHON) (12–14), possibly due to preservation of ipRGCs subserving the PLR (15). Previous reports have suggested that the pupillary reactions also could be spared in patients with autosomal dominant optic atrophy (ADOA), a finding supported by pupillographic, pathologic, and circadian-rhythm studies (15, 16). ADOA (17), the most common hereditary optic neuropathy (18), being due to a mutation in the OPA1 gene, causes irreversible bilateral visual loss because of retinal ganglion cell and nerve fiber atrophy (19–22). Earlier pupillometric studies in humans (23) and in animals (24) have indicated that the pupillary light reactions are preserved in ADOA. While studies on genetically confirmed ADOA have been performed on animal models, to the best of our knowledge, such studies on humans are lacking.

Consequently, the aim of the present study was to answer the question: is ipRGC function preserved in ADOA patients with genetically verified mutations? To do this, we examined the PLR (a NIF function), the retinal structure, and the visual field analysis (VFA) and the best corrected visual acuity (BCVA) (both visual functions) comparing them within the sample of patients and against a normal control sample.

## Materials and Methods

### Participants

Patients with *OPA1* ADOA, recruited from the National Danish Institute for the Visually Disabled (Department of Ophthalmology, Glostrup Hospital, University of Copenhagen, Department of Human Molecular Genetics, Glostrup, Denmark), had either the mutation c.983A > G (14 patients) or the mutation c.2708_2711delTTAG (15 patients) (25). These two mutations were also selected for a separate study of phenotype modifying factors (Rönnbäck et al., in preparation). Healthy controls were recruited by advertisements (26). The analysis excluded those, for whom one or more of the parameters needed for the analyses used in the present study were missing, thus leaving 40 controls. None of these control subjects exhibited any history or sign of ocular or systemic disease, nor used medication known to affect the PLR.

Exclusion criteria included high myopia (≤−6.0 diopters), glaucoma, cataract, other significant ocular or systemic conditions including arterial hypertension or diabetes mellitus, and use of medications affecting the PLR. After excluding 1 patient with dense cataract, we explored a population of 29 patients from 11 separate families, and 40 healthy controls without any history or signs of systemic or ocular pathology. ADOA patients and controls underwent a standard clinical eye examination, including determination of BCVA using the ETDRS protocol, slit-lamp examination, applanation tonometry, color vision testing (Farnsworth 15D and Ishihara’s test), fundoscopy, and fundus photography. High-definition spectral-domain optical coherence tomography (OCT) (Cirrus, software version 6.0, Carl Zeiss Meditec, Dublin, CA, USA) and automated VFA by SITA standard 30-2 (Humphrey Instruments, Type 750, CA, USA) were also performed. The average peripapillary retinal fiber layer thickness (RNFL) was computed by the OCT software, based on a 512*128 scan centered on the optic nerve, and the macular ganglion cell and inner plexiform layer (GCL), based on the 200*200 scan, centered on the foveola of the macula. Only eyes with signal strength ≥6 were included in the study; by convention, left eyes were analysed and compared in the ADOA group and among healthy controls. The study, which followed the rules of the Helsinki Declaration, was approved by the local ethics committee. Prior to written consent, each participant received relevant information relating to the experimental protocol.

### Pupillometry

The monochromatic pupillometer employed and the procedure used have been described in detail elsewhere (27). Briefly, the instrument consists of a LED light source, delivering either blue or red light of a defined wavelength and luminance for a predetermined time (usually 20 s) to one eye. An infrared system records the area of the contra-lateral pupil before, during, and after light stimulation. The two sections are synchronized, being controlled by a common computer program. The area of the contra-lateral pupil is monitored with a frequency of 20 Hz and converted into a diameter, assuming a circular pupil. Light intensity (luminance) was 300 cd/m^2^ for red and blue light, corresponding to 10^14,9^ quanta/cm^2^/s (red) and 10^14,8^ quanta/cm^2^/s (blue) and less for the infrared detecting system, preliminary studies showing 300 cd/m^2^ to be sufficient to saturate the PLR-generating system. All intensities were chosen well below the recommendations of ANSI-2007 and ICNIRP. Initial calibration was performed with the RP-655 spectrophotometer (Photo Research, Chatsworth, CA, USA). A baseline pupil diameter (BPD) was calculated as the mean diameter during 10 s in darkness, prior to light initiation. The pupillary diameter (PD), obtained during light-on and -off, was expressed relative to the BPD: PD/BPD, yielding the normalised PD, NPD. When light was projected into the stimulated eye, the PD decreased from BPD to the PD, i.e., BPD–PD, which, when normalized [(BPD − PD)/BPD] and summed from time = *t*_0_ to time = *t*_1_, was expressed as: $\sum_{t_{2}}^{t_{1}}\left(1.0-\mathrm{NPD}\right)$ ≡ Area under the curve (AUC_t0–t1_).

**Figure 1:**
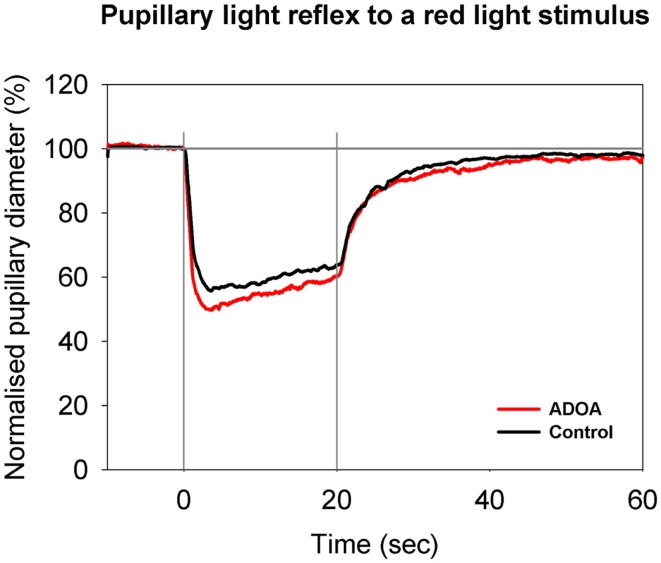
**Pupillary contraction to a red light stimulus (660 nm) as a function of time (s)**. A constant and continuous stimulus of 300 cd/m^2^ was applied at time 0 (first vertical gray line) and discontinued at the end of the 20th second (second vertical gray line). The stimulus was applied to one eye and the consensual, pupillary contraction of the other eye recorded. The red graph represents the mean of contractions set off by stimulation in ADOA eyes, and the black the mean of contractions set off by stimulation in control eyes. During light stimulation, contraction is larger in the red graph than in the black graph. When the light stimulus is terminated, fairly rapid re-dilatation ensues. No statistically significant difference is detected (cf. Table 2). The red graph shows the mean value from 29 subjects suffering from ADOA, the black graph the mean of 40 healthy control eyes. The AUC is the area between the horizontal line: NPD = 100% and the graph in question.

**Figure 2:**
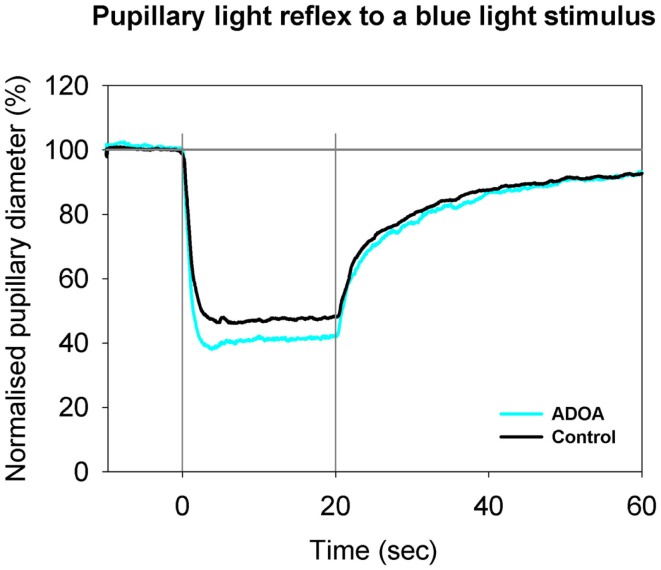
**Pupillary contraction to a blue light stimulus (470 nm) as a function of time (s)**. Time period, stimulus luminance, and size of input pupil as in Figure 1. The light blue graph represents the mean of pupillary contractions set off by stimulation of the ADOA eyes, and the black graph the mean of contractions set off by stimulation in control eyes. In comparison with the graphs in Figure 1, contraction is larger during light on in both graphs, and post-light stimulus re-dilatation far slower than that due to red light. After light termination, the difference between the two graphs is negligible. Results represent mean values from 29 subjects suffering from ADOA and from 40 healthy controls.

An AUC was calculated for each of three separate time-periods: (1) during exposure to light, i.e., during the 20 s of the illumination of the pupil (AUC_0–20 s_), (2) during the first 10 s of darkness after the light was turned off (AUC_20–30 s_), and (3) during the following 20 s of darkness, i.e., in the interval from 10 to 30 s after the light was turned off (AUC_30–50 s_). A large AUC indicated the presence of a small (constricted) pupil over the time-period considered (Table 2; Figures 1 and 2). Specific AUCs were calculated for exposure to blue light and to red light. The post-illuminatory pupillary response after exposure to 300 cd/m^2^ blue light was considered as a measure of ipRGC function (8), while the pupillary response during illumination must be generated by S-cones and ipRGCs. The response to red light during illumination was similarly considered as a measure of synaptic function, generated mainly by L-cones. Assuming the synaptic transmission by way of the ipRGCs to be similar for red and for blue light, the (AUC_blue light_ minus AUC_red light_) would be a measure, solely, of the intrinsic mechanism [*p*-values for this outcome measure are given in Table 2 (denoted p*)].

Pupillometry sessions were performed in a dark room, in which luminance was controlled by the investigator, as previously described (28). All sessions were performed between 9 a.m. and 4 p.m. One eye was exposed to light, as described below, and the pupil of the contra-lateral eye video filmed. While the patient was seated and the instrument adjusted, ambient light was mesopic for approximately 5 min. Then, prior to examination, the patient was exposed to darkness for 1 min. The pupillometry session was composed as follows: 10 s in darkness (measurement of the baseline pupil), then 20 s of exposure to red light (measurement of illuminatory response), and finally 60 s in darkness (measurement of the post-illuminatory response). After 5–7 min of rest, the entire session was repeated, this time with isoluminant (300 cd/m^2^) blue light.

### Statistical analysis

A normal distribution was assumed for age, baseline pupil area, RNFL and GCL thickness, for BCVA, and for pupillary responses (AUC), these data being expressed as mean and SD for each outcome measure. The BPD was calculated using the procedure described above. AUC data from ADOA patients and controls were compared by *t*-tests. Comparisons of age, RNFL thickness, GCL thickness, and BCVA were performed using unpaired *t*-tests, and of MD using the Mann–Whitney test. Correlations among RNFL thickness, BCVA, age, and AUC were performed using Pearson’s test, while for correlations with MD, Spearman’s test was used. A general linear model was used for the analysis of covariates of the AUC. A *p*-value below 0.05 was considered statistically significant. Analysis included only left eyes in ADOA patients and in controls. Calculations were performed using SAS statistical software (SAS version 9.3., SAS Institute Inc., Cary, NC, USA).

## Results

### Samples

Twenty-nine patients with genetically confirmed ADOA (7 men and 22 women) were included together with 40 healthy controls (22 men and 18 women). ADOA patients and controls were of comparable ages: ADOA patients were 18–72 years (mean 49.7), and controls 26–68 years (mean 44.7), *t*-test between groups, *p* = 0.177. Within the group of ADOA patients, no difference between the two eyes was detectable for any outcome measure (*t*-tests and Mann–Whitney test: individual *p*-values ranging from 0.10 to 0.95). Since the study comprised two genetically distinct samples, initially all outcome variables were compared by *t*-tests and by the Mann–Whitney test between samples. As the tests revealed no significant differences, with the exception of AUC_20–30 s_ to red light (for c.983A > G: 1.35, for c.2708_2711 delTTAG: 1.88; *p* = 0.03; *t*-test), they were pooled (*t*-tests between AUCs: individual *p*-values ranging from 0.0635 to 0.2592; all other outcome variables and age: *p*-values ranging from 0.56 to 0.95).

### Pupillary light reflex

The mean baseline pupil area in ADOA patients was 36.75 mm^2^, SD = 12.06, which was significantly smaller than the baseline pupil area in controls (*p* = 0.0006, Table 1). In ADOA patients, the mean baseline pupil area was negatively correlated with age (*p* = 0.0025, *R*^2^ = 0.29). When the AUCs were analysed in a general linear model, the baseline pupil area as well as age, gender, RNFL-, GCL-thickness, and MD were non-significant covariates for the outcome (*p* > 0.2, data not shown). Significant differences between AUC in ADOA and controls were found only for AUC_0–20 s_ to blue light, the former (ADOA) being the larger (Table 2; Figure 2), and for AUC_30–50 s_ after red light stimulation. Since, however, the preceding part of the PLR after red light stimulation (i.e., AUC_20–30 s_) was normal, this must be an artifact (Table 2; Figure 1).

**Table 1:** **Distribution of best corrected visual acuity (BCVA) as measured in the ETDRS system, baseline pupil area (Pupil), average macular ganglion cell and inner plexiform layer thickness (GCL), average peripapillar retinal fiber layer thickness (RNFL), mean deviation in visual field analysis (MD), and age of ADOA patients and controls**.

ADOA	BCVA age (ETDRS) years	Pupil (mm^2^)	GCL (μm)	RNFL (μm)	MD (dB)	Age (years)
Mean	56.48	36.75	51.72	61.79	− 4.37*	49.72
SD	23.74	12.06	7.89	9.44	5.01**	15.87
**Controls**
Mean	91.18	48.59	79.65	88.85	− 0.28*	44.67
SD	4.87	14.41	6.77	10.63	1.80**	14.64
***T******-test***
*p*	<0.0001	0.0006	<0.0001	<0.0001	<0.0001***	0.177

Since MD did not conform to a normal distribution, it is shown as median (*) and Inter Quartile Range (**), and compared by the Mann–Whitney test (***).

**Table 2:** **The pupillary light reflex, expressed as AUC, during (light on) and after (light off) stimulation with either blue (470 nm) or red (660 nm) light**.

AUC (s)	0–20 s light on		20–30 s light off		30–50 s light off	
Light	Blue	Red	Blue	Red	Blue	Red
**ADOA**
Mean	11.34	8.52	3.28	1.67	3.23	1.27
SD	1.42	2.26	0.88	0.68	1.8	0.88
**Controls**
Mean	10.24	7.8	3.14	1.57	2.88	0.78
SD	1.16	1.5	0.72	0.52	1.62	0.56
***T******-test***
*p*	0.0008	0.15	0.45	0.49	0.41	0.01
p* (blue–red)	0.35		0.22		0.70	

Apart from the values generated by blue cones and ipRGCs during blue light stimulation (AUC_0–20 s_), all blue values are non-significant. All values generated by red light are non-significant, apart from AUC_30–50 s_, which most likely is due to noise (see text). When red AUCs are subtracted from blue AUCs, “blue − red,” being a measure of intrinsic activity, is non-significant (p*), during all three time periods (see Materials and Methods).

Correlation was non-significant between AUC (irrespective of color of stimulant light) and the age of ADOA patients, and between AUC and average RNFL, apart from AUC_0–20 s_ to blue light, that is during illumination.

A significant correlation was detected between visual field MD and AUC_0–20 s_ to red and blue (*p* = 0.011 and 0.017, Spearman), but the post-illumination pupillary reaction, expressed as AUC_20–30 s_ was only significant after blue light illumination (*p* = 0.020, Spearman) in agreement with different response kinetics of cone pigments and melanopsin.

The other measure of visual function, BCVA, showed significant correlation between (AUC_0–20 s_) and (AUC_20–30 s_) to blue light (*p* = 0.006, *R*^2^ = 0.25 and *p* = 0.034, *R*^2^ = 0.16), but not to any other AUC.

Despite poor visual acuity (ETDRS = 56 letters), the post-illumination pupillary responses did not differ significantly from those of healthy controls (Table 2) and no statistical effect was noted of peripapillary RNFL thickness, ganglion cell-inner plexiform layer thickness, or age.

### Visual function and RNFL

As would be expected, the mean visual acuity was severely reduced in ADOA patients [mean BCVA (ETDRS) = 56.48 symbols, SD = 23.74], as compared with healthy controls [mean BCVA (ETDRS) = 91.18 symbols, SD = 4.87, *t*-test, *p* < 0.0001, Table 1]. A mean visual acuity of 56.48 symbols in the ETDRS notation corresponds, roughly, to 0.25 Snellen vision. Likewise, the visual field MD values were significantly reduced in ADOA patients (median −4.37 dB, IQR = 5.01) as compared with controls (median −0.28 dB, IQR = 1.8, Mann–Whitney, *p* < 0.0001, Table 1). BCVA was significantly correlated with MD (*p* < 0.0001).

In ADOA eyes, the mean average peripapillary RNFL thickness was 61.79 μm, SD = 9.44, and the mean average GCL thickness 51.72 μm, SD = 7.89. These values were significantly lower than those recorded in controls (Table 1), in whom the mean average RNFL thickness was 88.85 μm (*t*-test, *p* < 0.0001), and the mean average GCL thickness 79.65 μm (*t*-test, *p* < 0.0001).

## Discussion

### Pupillary light reflex

The main finding of the present pupillometry study on a genetically confirmed ADOA population is the preserved post-illumination pupillary responses, indicating normal function of the ipRGCs, despite a marked loss of vision, decrease of MD, and of GCL and RNFL thickness. Earlier studies, notably the pioneering study of Bremner et al. performed before the discovery of the ipRGCs in humans, did indicate that in ADOA there was dissociation between the relatively preserved pupillary responses and the altered visual function (23). Another, more recent study reported similar findings (16) in eight patients with a clinical diagnosis of Hereditary Optic Neuropathy. Interestingly, in an *OPA1* mutant mouse model, the NIF functions, subserved by the anatomically intact ipRGCs, were also preserved, including the circadian behavior and the pupillary light response (24). Taken together, these studies suggest that in ADOA, there is a functional preservation of the network originating in the ipRGCs in the retina (15).

The factors contributing to resistance of ipRGCs to neurodegeneration are not known. It has been speculated that these cells may have lower energy demands than other retinal ganglion cells, and therefore, were spared the mitochondrial dysfunction (24). The presence of many mitochondria in ipRGCs (29) would, however, argue against it. Among the factors that have been involved in ipRGCs robustness is pituitary adenylate cyclase activating polypeptide (PACAP), which is expressed in ipRGCs (30). It is also possible that this system, which is crucial for NIF functions, may have an intrinsic resistance to ophthalmic injuries (31), in this case, resistance to short term ischemia, arguing against the implications of many mitochondria in ipRGCs and supporting the case for resistance to ischemia because of a low metabolic rate. The observation that the *trans*-form of melanopsin may be regenerated to the *cis*-form by long wave light (32), circumventing the usual metabolic pathways, is in agreement with this observation. In the present study, we observed no difference between the AUC to red light in ADOA and controls, neither during light-on nor after. Since the PLR to red light is solely generated in red light sensitive cones, this lack of difference is only explainable by the presence of healthy bipolar and amacrine cells synaptically transmitting impulses to the ipRGCs. From this point of view, one could argue that the ipRGC system is not inordinately resistant to trauma [cf. (10)], but rather that it is the RGC system in ADOA which is failing (33).

### Methodological issues

The control population was examined in 2009, the ADOA sample in 2013. Could this time gap bias the results, especially since baseline pupil area was significantly larger in controls than in ADOA patients (Table 1)? We may split this question into two. First, could the time gap *per se* be an important bias? Since the selection and the sampling procedure were not changed over time, and the general population characteristics must be considered constant during these 4 years, taking into account the slowness of evolution in man and the stable living conditions in Denmark, a sample drawn in 2013 should not differ significantly from one drawn at random 4 years earlier (cf. also outcome measures in Table 1). Hence, the time gap *per se* is not likely to be an important bias, if any. Second, procedures and instrumentation might have changed over the years. This, however, is not so: time of the day, facilities used, including location, room, instruments, etc., were all the same as in the ADOA examination. The output of the pupillometer itself was stable, and as a precaution calibrated before each session, or at least thrice daily. The area of the pupil was constantly monitored by the examiner and registered and stored by the pupillometer in real time, so that any change or aberration would be immediately apparent. The pupillometer was neither modified or changed nor did it ever break down. Hence, we feel justified in using this control sample in the present study. Finally, neither the AUCs of the controls (*p* = 0.3–0.9, determination coefficients <2.6%) nor those of the ADOA patients were significantly correlated to baseline pupil area. We have to conclude that the baseline pupil area in controls was indeed larger than that in ADOA, and that this difference is unlikely to be due to the time gap itself or to conditions derived from it. How, then should this difference be explained? The number of photons initially entering the eye must be proportional to the area of the pupil. Thus, even though controls eyes, at least initially, receive more photons than ADOA eyes, the PLR of the latter is equal to or larger than that of the former. The present setup does not enable us to explain why absolute pupil area is the larger in controls and how this is achieved, but two conclusions may safely be drawn: the regulator is either supra-nuclear or post-pupillar, most likely both, and the area of the baseline pupil is not the rate-limiting step for the PLR. Could it be that the light-induced constriction of the pupil is part of an opto-endocrinological system, controlled by negative feedback? If this is so, then it readily explains why maximal pupil size is not a significant factor in the regulation of the PLR. One might argue that the important thing is not that the maximal amount of light enters the eye, but that sufficient light does. This could be the function of the light-induced PLR.

In conclusion, the present pupillometric study indicates that in genetically confirmed ADOA, the PLR is preserved and similar to that observed in healthy controls, and does not decrease with age. The synaptic as well as the intrinsic function of the ipRGCs are preserved, contrasting with the profound visual loss and diffuse retinal ganglion cell atrophy, so characteristic of ADOA.

## Conflict of Interest Statement

The authors declare that the research was conducted in the absence of any commercial or financial relationships that could be considered as a potential conflict of interest.
